# The effects of Various Endodontic Irrigants on the Push-out Bond Strength of Calcium-Enriched Mixture Cement and Mineral Trioxide Aggregate

**DOI:** 10.22037/iej.2016.5

**Published:** 2016

**Authors:** Safoora Sahebi, Fereshte Sobhnamayan, Sina Naghizade

**Affiliations:** a *Department of Endodontics, Dental School, Shiraz University of Medical Sciences, Shiraz, Iran*

**Keywords:** Bond Strength, Calcium-Enriched Mixture Cement, Irrigants, Mineral Trioxide Aggregate, Push-Out

## Abstract

**Introduction:**

The aim of this *in vitro *study was to evaluate the effect of various irrigants on the push-out bond strength of calcium-enriched mixture (CEM) cement and mineral trioxide aggregate (MTA).

**Methods and Materials:**

A total of 140 dentin disks with a thickness of 1.5±0.2 mm and lumen size of 1.3 mm, were randomly divided into 12 groups (*n*=10) and 4 control groups (*n*=5). The lumen of disks in groups 1, 2, 3, 7, 8, 9 were filled with CEM and groups 4, 5, 6, 10, 11, 12 were filled with MTA. Control groups were filled with CEM and MTA. Specimens were incubated at 37^°^C for one day in groups 1 to 6 and seven days in groups 7 to 12. After incubation the samples were divided into three subgroups (*n*=10) that were either immersed for 30 min in 5.5% sodium hypochlorite (NaOCl), 2% chlorhexidine (CHX) or saline solution. The push-out bond strength values were measured by using a universal testing machine. The nature of the failures were determined by light microscope. Data was analyzed using the three-way ANOVA to evaluate the effect of material type, different irrigants and time intervals. Post hoc Tukey’s test was used for two-by-two comparison of the groups.

**Results:**

CEM cement significantly showed a higher push-out bond strength in comparison with MTA (*P*=0.001). The elapse of time significantly increased the bond strength (*P*=0.001). There was no significant difference between the irrigants used in this study (*P*=0.441). Bond failure was predominantly of mixed type in MTA and of cohesive type in CEM samples.

**Conclusion:**

Based on this study, endodontic irrigants did not influence the push-out bond strength of MTA and CEM cement.

## Introduction

Root perforations are responsible for approximately 9.6% of endodontic failures [1]. Perforation of the cervical area and furcations show poorer prognosis as compared with those in other root areas [2-4]. Perforation repair materials should be dimensionally stable, well tolerated by periradicular tissues, provide a proper seal, have good adaptation with the perforated wall areas and be unaffected by the presence of moisture [5]. 

Mineral trioxide aggregate (MTA) and calcium-enriched mixture (CEM) cement are being used as root perforation repair materials because of their excellent biocompatibility, superior sealing ability, hard-tissue induction, cementogenesis and PDL formation and their ability to set in the presence of a wet environment and also blood contamination [6-14]. Considering the clinical applications of these two materials, the bond strength is an important factor in providing a favourable seal between the root canal system and the external surface of the root. Therefore, these materials should resist the dislodgement forces such as functional forces and forces resulting from the placement of restorative materials. Push-out bond strength test is a valuable technique for the evaluation of this kind of bond [15-19].

Previous studies have reported different treatment strategies and sequences used for sealing the root and furcation perforations with MTA [20, 21]. One of these strategies is to place the perforation sealing material into the perforation site after complete instrumentation and obturation of the canals [22]. The deficits of this treatment sequence are as follows: firstly, some irrigants that are used during the cleaning and shaping of the root canals may cause irritation of periodontal tissue in the perforation site. Secondly, the obturation materials can egress through the perforation during compaction and thirdly, the root canal space might be contaminated by the ingress of contaminated tissue fluids containing even bacteria from the perforation site [23, 24].

Therefore, a wise clinician should immediately repair the furcation perforations in order to minimize the bacterial contamination and periodontal tissue irritation [25].

During the repair of perforation and endodontic treatment, various irrigants such as chlorhexidine gluconate (CHX), sodium hypochlorite (NaOCl) and normal saline might be used to clean the root canal system [26]. This process causes an unavoidable contact of irrigants with the perforation repair material. This can be avoided by postponing the treatment several days after perforation repair in order to have the initial set of the repair material. 

There is no information about the effects of different irrigation solutions on the push-out bond strength of CEM cement after perforation repair. Therefore, the aim of this *in vitro *study was to evaluate the effect of various irrigants on the push-out bond strength of CEM cement and MTA.

## Materials and Methods

In this *in vitro* study, 80 freshly extracted, single-rooted human teeth including 50 maxillary central incisors and 30 mandibular premolars with mature apices and intact roots were selected and stored in 0.5% chloramine-T before use. Teeth with cracks or internal resorption were excluded from the study. The crowns of all teeth were removed by using a diamond disk. The middle third of the roots were sectioned perpendicular to the long axis with a diamond saw microtome (Mecatom T180; Presi SA, Angonnes, France) to obtain 140 dentin disks with a thickness of 1.5±0.2 mm. The lumens of the dentin disks were enlarged with #2 to 5 Gates Glidden drills (Dentsply Maillefer, Ballaigues, Switzerland) to achieve a diameter of 1.3 mm. To remove the smear layer, we immersed the disks in 17% ethylenediaminetetraacetic acid (EDTA, Endo-Solution, Cerkamed, Poland) and then in 2.5% sodium hypochlorite (Chloraxid, Cerkamed, Poland) for 3 min each [27]. The samples were then immediately washed in distilled water and dried. The dentin disks were randomly divided into 12 groups (*n*=10) and four control groups (*n*=5). CEM cement (BioniqueDent, Tehran, Iran) was mixed according to the manufacturer’s instructions. The materials were incrementally placed in lumens of slices and condensed on a glass slab. Excess material was trimmed from the surface of the dentine disks with scalpel. The lumen of groups 1, 2, 3, 7, 8 and 9 were filled with CEM cement. MTA (Angelus, Londrnia, PR, Brazil) was mixed at a powder to liquid ratio of 3:1. The lumen of groups 4, 5, 6, 10, 11 and 12 were filled with MTA. Two control groups were filled with CEM cement and two with MTA. The samples were wrapped in wet pieces of gauze, placed in an incubator and allowed to set at 37^°^C with 100% humidity. Specimens were incubated for one day (groups 1 to 6) and seven days (groups 7 to 12). Control groups were incubated in the same situation for one and seven days. Immediately after incubation the samples were divided into three subgroups (*n*=10) to be immersed in 5.25% NaOCl (Chloraxid, Cerkamed, Poland), 2% CHX (Gluco-chex, Cerkamed, Poland) or saline solution. After 30 min of immersion, all samples were removed from the test solutions and rinsed with distilled water. While in control groups, a wet piece of gauze was placed over each test material without any irrigation (*n*=5).

The push-out bond strength values were measured by using a universal testing machine (Zwick/Roell, Z050; Zwick/Roell, Ulm, Germany). Dentin disks were placed on a metal slab with a central hole to allow free movement of the plunger. The MTA and CEM cement was loaded with a 0.7 mm diameter cylindrical stainless steel plunger at a speed of 1 mm/min. The maximum load applied to materials before dislodgement was recorded in Newton’s. To express the bond strength in megapascals (MPa), recorded values in Newton’s were divided by the adhesion surface area of MTA and CEM cement in square mm calculated according to the following formula [27]: (N/2*πrh*), where *π* is the constant 3.14, *r* is the root canal radius and *h* is the thickness of the root slice in mm. 

The slices were then examined by light microscope (Dino-Lite, Tai-pei, Taiwan) at 40× magnification to determine the nature of the bond failure. Each sample was categorised into one of three failure modes: adhesive failure at the material and dentin interface, cohesive failure within material and mixed-failure mode.

Multivariate analysis of variance was used to evaluate significance of the effect of material type, different irrigants and time intervals. The post hoc Tukey’s test were used for the two-by-two comparison of the groups. Statistical significance was defined at 0.05. SPSS software (SPSS version 18.0, SPSS, Chicago, IL, USA) was used for the analysis of data.

## Results

No significant interaction effect has been found between the three variables (material type, different irrigants and time intervals) (*P*=0.887). The only significant interaction effect was found between time interval and the material that has been used (*P*=0.002). The highest (1.8 MP) and the lowest (0.58 MP) bond strength values were recorded in groups CEM/control at seven days and MTA/normal saline at 24 h, respectively (Table 1). Regardless of time and the irrigants used, CEM cement significantly showed a higher push-out bond strength in comparison with MTA (*P*=0.001). Regardless of the irrigants and the material used, the elapse of time significantly increased the value of bond strength (*P*=0.001).

There was no significant difference between the irrigants used in this study (*P*=0.441).

**Figure 1:**
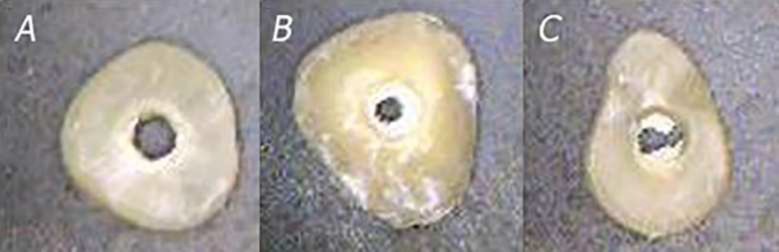
Various failure modes of samples under 40× magnification stereomicroscope; *A)* Adhesive failure; *B)* Cohesive failure within the material; *C)* Mixed failure

Tukey’s post hoc test showed that in seven-day samples CEM cement significantly had higher bond strength than other groups (Table 2). Bond failure was predominantly of mixed type in MTA samples and of cohesive type in CEM cement samples, although some samples showed other types of bond failures (Table 3) (Figure 1). 

**Table 1:** Mean (SD) of push-out bond strength in test groups

**Material**	**Irrigants**	**Time (days)**	**Number**	**Mean (SD)**
**CEM**	**Control**	1	5	0.78 (0.27)
		7	5	1.81 (0.70)
	**CHX**	1	10	0.94 (0.26)
		7	10	1.66 (0.66)
	**NaOCl**	1	10	0.71 (0.32)
		7	10	1.63 (0.75)
	**Saline**	1	10	0.76 (0.26)
		7	10	1.68 (0.93)
	**Total**	1	35	0.80 (0.28)
		7	35	1.68 (0.74)
**MTA**	**Control**	1	5	0.89 (0.52)
		7	5	1.14 (0.64)
	**CHX**	1	10	0.77 (0.45)
		7	10	1.09 (0.69)
	**NaOCl**	1	10	0.69 (0.27)
		7	10	0.87 (0.70)
	**Saline**	1	10	0.58 (0.36)
		7	10	0.91 (0.46)
	**Total**	1	35	0.71 (0.39)
		7	35	0.99 (0.61)

**Table 2:** Pairwise analysis of push-out bond strength in different time intervals (*P*<0.05

**Material/Time (I)**	**Material/Time (J)**	**Mean (SD) of difference (I-J)**	***P*** **-value**
**CEM/1 day**	**CEM/7 days**	-0.87 (0.12)*	0.001
	**MTA/1 day**	0.09 (0.12)	0.898
	**MTA/7 days**	-0.18 (0.12)	0.492
**CEM/7 days**	**CEM/7 days**	0.87 (0.12)*	0.001
	**MTA/1 day**	0.96 (0.12)*	0.001
	**MTA/7 days**	0.69 (0.12)*	0.001
**MTA/1 day**	**CEM/1 day**	-0.09 (0.12)	0.898
	**CEM/7 days**	-0.96 (0.12)*	0.001
	**MTA/7 days**	-0.27 (0.12)	0.154
**MTA/7 days**	**CEM/1 day**	0.18 (0.12)	0.492
	**CEM/7 days**	-0.69 (0.12)*	0.001
	**MTA/7 days**	0.27 (0.12)	0.154
			

**Table 3:** Failure modes (%) of each test material

**Groups (N)**	**Failure mode (Adhesive/Cohesive/Mixed)**
**CEM (70)**	10/71.5/18.5
**MTA (70)**	15.7/22.8/61.5

## Discussion

This *in vitro* study compared the bond strength of MTA and CEM cement when exposed to different irrigants (hypochlorite 5.25%, chlorhexidine 2% and normal saline) in two different time intervals (one and seven days).

The present study showed that different irrigants did not have a significant effect on the push-out bond strength of MTA and CEM cement. This finding is in accordance with that of a recent study on the push-out bond strength of MTA when mixed with CHX, which showed that mixing MTA with CHX does not have an adverse effect on the push-out bond strength of this material [28].

Ping Yan *et al. *[26] also showed that the bond strength of MTA-dentin in contact with 2% CHX did not show a significant decrease. On the other hand, Guneser *et al. *[29] showed that exposure of MTA to 2% CHX after only 10 min of setting significantly decreased the push-out bond strength of this material. Hong *et al. *[30] also showed that 2% CHX reduced the push-out bond strength of accelerated MTA after 10 min of initial setting. Nandini *et al. *[31] also showed that 2% CHX decreased the surface hardness of MTA and suggested that irrigation with CHX is better to be postponed to 24 h after MTA setting. This difference between the result of the present study and the aforementioned ones could be attributed to the different time intervals. The present study exposed the materials to different irrigation solutions after 24 h but the previous ones exposed them after just 20 min.

Hypochlorite-treated samples in the present study resisted dislodgement forces just as in other groups. This finding was in accordance with Guneser *et al. *[29] who showed the effect of NaOCl on the push-out bond strength of MTA was not significant. Ping Yan *et al. *[26] stated that although the bond strength of MTA-dentin showed a decreased tendency in the 5.25% NaOCl group, it was not significantly different with control group. They also showed the microstructure on the interfacial layer of dentin walls in the NaOCl group was similar to those of control group.

Hong *et al. *[30] showed that NaOCl-treated accelerated MTA groups showed significantly higher push-out bond strength than CHX-treated groups. Some other studies also showed NaOCl might have a positive effect on the push-out bond strength of MTA [25, 32]. 

In the present study, saline-treated MTA samples resisted dislodgment forces nearly equivalent to other irrigants, which is not in accordance with the results of the study by Loxely *et al. *[25] who indicated that the compressive strength of MTA increased when immersed in saline solution for seven days because of the remaining unreacted mineral oxides. These may be solidified after additional supplied hydration and may result in the increased strength of material. This difference could be attributed to the time of exposure of these materials to normal saline, as in the present study the samples were exposed to the irrigants for just 30 min not for seven days.

CEM cement also did not show any significant decrease in the presence of different irrigants. A recent study on CEM cement showed mixing CEM cement with 2% CHX has an adverse effect on the push-out bond strength of this cement [27]. This difference could be attributed to the different set-up systems used in these studies as, in the present study; the samples were exposed to 2% CHX after 24 h of setting.

Increasing the setting time from 24 h to seven days increased the bond strength of MTA and CEM cement. Gancedo-Caravia and Garcia-Barbero [18] showed that with the lapse of time from three to 21 days under wet conditions, the push-out bond strength of MTA showed a significant increase. Torabinejad *et al. *[33] showed that the compressive strength of MTA increased after 21 days of immersion in water. Another study comparing the bond strength of MTA using anaesthetic solution indicated the bond strength of MTA increased from 24 to 72 h [34].

Richard *et al. *[3] in a study on the effect of blood on retention characteristics of MTA showed the 72-h samples displayed significantly greater resistance to displacement than the 24-h samples, and the seven-day samples displayed significantly greater resistance to dislodgement forces than the 24- and 72-h samples.

Rahimi *et al. *[35] also showed the bond strength of CEM cement mixed with normal saline increased with the elapse of time from 24 h to seven days. A recent study on the effect of CHX on the push-out bond strength of CEM cement showed the mean bond strength after 21 days was significantly greater than that after three days [27].

Based on the results of present study, it is better to complete the root canal therapy one week after repair of perforation. In the present study, CEM cement showed significantly higher bond strength after 7 days in comparison with MTA. In contrast, Adl *et al. *[36] revealed that CEM cement had lower bond strength after 3 days compared to MTA. This difference can be attributed to the different time intervals in both studies. In another study, bond strengths of MTA and CEM cement to root-end cavities were statistically similar. Bond strengths of these materials in ultrasonically prepared cavities were higher than laser-prepared cavities [37].

In the present study, the bond failure in the MTA groups was of a predominantly mixed type, although some samples exhibited cohesive and adhesive failures. This is consistent with the results of the study by Rahimi *et al. *[35]. Guneser *et al. *[29] showed the bond failure in the MTA group was mostly of an adhesive and mixed type, which is relatively in agreement with the present study. 

In contrast, Adl *et al. *[36] showed that the bond failure of MTA was mostly of adhesive type, which is not in accordance with the present study.

In the study by Vanderweele *et al. *[3], the predominant type of bond failures was the adhesive type (MTA-dentin gap). These studies were not in agreement with the present study which could be attributed to the different environmental factors and brand of MTA (Angelus MTA) that have been used in the present study.

Results of this study showed the bond failure in CEM cement was mostly of cohesive type. Although Rahimi *et al. *[35] indicated the bond failures of CEM cement were mainly of mixed type.

Another study showed the failure mode of MTA and CEM cement was mostly of cohesive type [38], which is partly in agreement with the present study.

Sobhnamayan *et al. *[39] showed bond failure of CEM cement in the presence of a modulated acidic environment is mostly of cohesive and mixed type, which is also partly in accordance with the present study. However, the failure mode of MTA was mostly adhesive type in the presence of acidic and alkaline environments [16, 19].

The bond failure of CEM cement in the normal pH environment, in the presence of alkaline pH and also when mixed with CHX, is also predominantly of cohesive type, which is consistent with the present study [27, 36, 40].

## Conclusion

In conclusion, endodontic irrigants did not influence the push-out bond strength of MTA and CEM cement. The push-out bond strength of CEM cement was significantly greater than MTA. Increasing the incubation time significantly improved the bond strengths of all materials. Based on the result of our study, when repairing perforations in root canals, it is advisable to complete the second session of treatment after 7 days.
